# Assembling the Healthopolis: Competitive city‐regionalism and policy boosterism pushing Greater Manchester further, faster

**DOI:** 10.1111/tran.12421

**Published:** 2020-12-06

**Authors:** Colin Lorne, Anna Coleman, Ruth McDonald, Kieran Walshe

**Affiliations:** ^1^ Geography, Faculty of Arts and Social Sciences The Open University Milton Keynes UK; ^2^ Division of Population Health, Health Services Research and Primary Care University of Manchester Manchester UK; ^3^ Alliance Manchester Business School University of Manchester Manchester UK

**Keywords:** competitive city regionalism, devolution, Greater Manchester, National Health Service, policy assemblage, policy boosterism

## Abstract

Health and care policy is increasingly promoted within visions of the competitive city‐region. This paper examines the importance of policy boosterism within the political construction of city‐regions in the context of English devolution. Based on a two‐year case study of health and social care devolution in Greater Manchester, England, we trace the relational and territorial geographies of policy across and through new “devolved” city‐regional arrangements. Contributing to geographical debates on policy assemblages and city‐regionalism, we advance a conceptual framework linking crisis and opportunity, emulation and exceptionalism, and evidence and experimentation. The paper makes two key contributions. First, we argue health and care policy is increasingly drawn towards the logic of global competitiveness without being wholly defined by neoliberal political agendas. Fostering transnational policy networks helped embed global “best practice” policies while simultaneously hailing Greater Manchester as a place beyond compare. Second, we caution against positioning the city‐region solely at the receiving end of devolutionary austerity. Rather, we illustrate how the urgency of devolution was conditioned by crisis, yet concomitantly framed as a unique opportunity by the local state harnessing policy to negotiate a more fluid politics of scale. In doing so, the paper demonstrates how attempts to resolve the “local problem” of governing health and care under austerity were rearticulated as a “global opportunity” to forge new connections between place, health, and economy. Consequently, we foreground the multiple tensions and contradictions accumulating through turning to health and care to push Greater Manchester further, faster. The paper concludes by asking what the present crisis might mean for city‐regions in good health and turbulent times.

## Abstract 2

Health and care policy is increasingly promoted within competitive city‐region visions. Based on a two‐year study of health and social care devolution in Greater Manchester, England, we demonstrate how the “local problem” of governing health and care amid austerity politics was rearticulated as a “global opportunity” to forge new connections between place, health, and economy. The paper foregrounds the multiple tensions and contradictions accumulating through turning to health and care to push the city‐region further, faster.

## 1 ASSEMBLING THE HEALTHOPOLIS

In February 2015, news broke of an “unprecedented devolution deal” suggesting the city‐region of Greater Manchester, England, would take control of its entire £6bn health and social care annual budget (Manchester Evening News, 2015). The announcement came as a surprise to all but a select coalition of English National Health Service (NHS) and local government managers and political leaders who privately negotiated the deal with UK central government. The deal for health and social care built on a separate devolution agreement in 2014 giving increased controls over housing, planning, transport, skills, and policing to the Greater Manchester Combined Authority on condition of electing a new city‐regional mayor and increasing fiscal self‐sufficiency (Haughton et al., 2016). Health and social care devolution would prove politically contentious. Trade unions expressed concern over the speed of the deal and the absence of public scrutiny and local democracy. Then‐Labour Party Shadow Secretary of State for Health, Andy Burnham (later to become the first Mayor of Greater Manchester) cautioned devolution would create a “Swiss cheese” two‐tier NHS. Yet for Sir Howard Bernstein, long‐standing Chief Executive of Manchester City Council, embedding health and social care within the city‐region's wider devolution agenda generated “ground breaking opportunities that we can't afford to miss” (HSJ, 2015, np). Aligning health and care to their entrepreneurial vision as a global city‐region offered the potential, Bernstein assured, for health innovation to bolster efforts for Greater Manchester to become the best place in the world to live, work, and invest.

An “admiring gaze” rapidly took hold (Peck & Theodore, 2015, p. xxiv). Policy commentators, think‐tanks, and local elites were quick to herald the city‐region as a pioneer unifying economic growth with public service reform. Located in North West England, Greater Manchester already occupied a privileged position within the UK government's “Northern Powerhouse” agenda, a spatial strategy to manage regional uneven development by combining agglomerative economic growth with implementation of austerity measures (Jones, 2019). The think‐tank Reform promptly published the manifesto “Letting Go: How English Devolution can help solve the NHS care and cash crisis” (Warner & O'Sullivan, 2015). While notionally about England, its authors focused principally on Greater Manchester. With the city of Manchester at its heart, the prize on offer, so their argument went, was to become Healthopolis. Evoking Manchester's 19th‐century legacy as Cottonopolis, the city‐region could now fix what they deemed a failing health service that was no longer affordable by capitalising on the “industrialisation of good health” through devolution (Warner & O'Sullivan, 2015). The Chief Executive of Manchester's inward investment agency, MIDAS, would later promote Greater Manchester as a one of a kind test‐bed for innovation, arguing the scale of integration across the city‐region's health and care system offered unrivalled access for MedTech and life sciences companies. While academics cautiously framed the project the “Greater Manchester experiment” (Walshe et al., 2016), Greater Manchester – or rather, Manchester – was hailed as a distinctly *global* trailblazer, a place seemingly beyond compare: “The hope is that, once again, as Sir Robert Peel proudly claimed: ‘What Manchester thinks today, the world does tomorrow’” (Warner & O'Sullivan, 2015, p. 38).

The political construction of globally connected city‐regions has been the focus of much geographical interest in recent years (for instance, Brenner, 2019; Harrison, 2007; McGuirk, 2007; Scott, 2001). Prevailing political‐economic strategies have targeted the spatial reconfiguring of metropolitan regions towards what Ward and Jonas term “competitive city‐regionalism” (2004, p. 2119), amid the positioning of city‐regions as the “ideal scale for policy intervention in a globalized world” (Rodriguez‐Pose, 2008, p. 1029). Here, triumphalist economists have gained particular currency championing “success stories” of US‐inspired agglomerative growth encouraging city‐regions to open themselves up to attract as much global investment as possible on the promise of the “trickle‐down” benefits (Etherington & Jones, 2016). Neoliberal growth strategies adopting theories of agglomeration have rapidly become policy orthodoxy within nationally coordinated city‐regional projects in a renewed search for international competitiveness (Beel et al., 2016; Harrison & Hoyler, 2014). Indeed, might the ascendency of competitive city‐regions even mark a decisive moment in global capitalism? As Jonas and Moisio caution, geopolitical processes of “internationally‐orchestrated city regionalism” need to be examined within and beyond the spaces and strategies of national state institutions to understand how city‐regional actors, institutions, and spatial imaginaries are enrolled into new forms of statecraft, experimentation, and problem‐solving (2018, p. 17). More than solely focusing on the politics of accumulation, research into the ongoing construction of competitive city‐regions requires paying attention to the geographies of social reproduction, distribution, and different kinds of political struggles and conflicts (McGuirk, 2007; Ward & Jonas, 2004).

We pursue this line of inquiry through focusing on policy boosterism as a vector of competitive city‐regionalism, based on a two‐year study of health and social care devolution in Greater Manchester.^1^ Building on insights from geographical literature on policy mobilities and entrepreneurial place‐marketing, “policy boosterism” has been identified as a phenomenon whereby localised policy‐making helps facilitate inter‐urban competition by promoting places in a global race to become the “greenest,” “coolest,” or simply, “the best” (McCann, 2013). Accordingly, continuous political work is required to negotiate the tensions between extrospective place‐branding activities in pursuit of “global” competitiveness and the introspective dimensions of building and sustaining “local” pride and support. Expanding on research examining the role of health and health innovation within the reinvention of cities and regions (Sparke, 2011; Waldby, 2009; Wong & Bunnell, 2006), we demonstrate how health and care policy is increasingly drawn towards narratives of global competitiveness, without being wholly defined by neoliberal political agendas. Doing so, the paper addresses missing links in city‐regional research identified by Beel et al. (2016) by establishing how particular policy narratives are mobilised in the remaking of competitive city‐regions reworking what is left of the Keynesian welfare state.

We argue efforts to resolve the “local problem” of governing health and care under austerity have been rearticulated as a “global opportunity” to forge new connections between place, health, and economy. Contributing to scholarship on competitive city‐regions and policy assemblages (Baker & McGuirk, 2017; Savage, 2019), we seek to move beyond something of an impasse in recent debates by tracing the tensions between the relational and territorial geographies of policy across and through the new “devolved” city‐regional arrangements. We do so by advancing a conceptual framework linking crisis and opportunity, emulation and exceptionalism, and evidence and experimentation. Our study foregrounds how entrepreneurial local state managers negotiated a more fluid “politics of scale” (Allen & Cochrane, 2007) reworking their “unique opportunity” to align health and care with aspirations of becoming a globally competitive city‐region. Fostering ostensibly transnational learning networks helped embed “global best practice” policies while simultaneously positioning the city‐region as a place beyond compare. We show how policy boosterism helped promote the city‐region as the biggest experiment in health and care on a global scale to attract life sciences research and pharmaceutical companies into the city of Manchester. Yet with the devolution project following an evidence‐base dominated by the logics of “trickle down” agglomerative economic growth, we emphasise the conflicts with aims to foster city‐regional “inclusiveness” through linking health, wealth, and well‐being.

The paper unfolds as follows. We first outline tensions within the geographies of “fast policy” (Peck & Theodore, 2015) relating to health and welfare reforms, experimental statecraft, and the role of health and biomedicine within the reinvention of “global” cities and regions. We then situate health and social care devolution within the remaking of Greater Manchester before elaborating on our research methods “following the policy.” Tensions between crisis and opportunity, emulation and exceptionalism, and evidence and experimentation are examined across three sections, establishing links between policy boosterism and competitive city‐regionalism. Foregrounding the multiple forces, tensions, and contradictions accumulating through turning to health and care to push Greater Manchester further, faster, we conclude by asking what the global pandemic might mean for competitive city‐regions in good health and turbulent times.

## 2 FAST POLICY IN CRISIS AND GOOD HEALTH

The political sociologist Claus Offe once remarked on a paradox whereby “capitalism cannot coexist *with*, neither can it exist *without*, the welfare state” (1984, p. 153, original emphasis). The construction of the post‐war Keynesian welfare state was positioned as a solution to societal contradictions in the global north: bound up with (post)colonial exploitation and racial exclusions, Keynesian economic policies organised primarily at the national scale sought to secure near full employment through demand‐side management, stabilised through welfarist social policies aimed towards spatial re‐distribution, social reproduction of labour‐power through collective provision in areas such as housing, education, and healthcare, and accepting the collective bargaining power of trade unions (see further, Bhambra & Holmwood, 2018; Jessop, 1999; Offe, 1984). The NHS became a prominent feature of the British welfare state as a comprehensive universal public healthcare service funded through general taxation. It was symbolic of the changing balance of forces, with Labour Minister for Health Aneurin Bevan declaring, “no society can legitimately call itself civilised if a sick person is denied medical aid because of lack of means” (1952, p. 100).

As the contradictions of the Atlantic Fordist regime of accumulation and its co‐existence with the welfare state manifested themselves in various crises in the 1970s, social democratic consensus broke down, with welfare increasingly targeted for market‐orientated reform (Jessop, 2002). A series of regulatory experiments shifted towards attracting global capital, privileging inter‐urban competition, marketisation, and welfare retrenchment (Brenner, 2019). Any dominance of the national scale was unsettled, with social policy increasingly subordinate to economic policy, alongside the discrediting and dismantling of Keynesian‐welfarist institutions. While the NHS continues to hold political and emotional significance across Britain, contemporary spatial configurations of health and care have been heavily impacted by the restructuring of welfare services, marketization, and the financialisation of care (on the latter, see further Horton, 2019).^2^

There is now burgeoning geographic scholarship concerned with the rise of “fast policy,” characterised by accelerated worlds of globally circulating policies mobilised by an ever‐expanding cast of intermediaries promoting “quick‐fix” solutions to various crises (Peck & Theodore, 2015). Accordingly, critical policy mobilities literatures take seriously the social and political dimensions of policies‐on‐the‐move paying close attention to the circulation of “best practice” policy models, knowledge, and expertise (Temenos & McCann, 2013). Here, we can trace connections between policy and place through the logics of competitiveness and place‐marketing such that “policy boosterism” can be understood as an extension of urban entrepreneurialism (Harvey, 1989). According to McCann, policy boosterism is a “subset of traditional branding and marketing activities that involves the active promotion of locally developed and/or locally successful policies, programs, or practices across wider geographical fields as well as to broader communities of interested peers” (2013, p. 5). Becoming identified as a policy “exemplar” can therefore become strategically helpful in fostering new growth opportunities (Temenos & McCann, 2013).

Yet, a strange tension exists between policy competition, whereby the hottest policies present new investment and promotional opportunities (Peck & Tickell, 2002), and collaboration among intermediaries participating in policy sharing, learning, and reflection (McCann, 2013). The search for the latest model to emulate comes up against a desire for cities to proclaim their exceptionalism, as seemingly unlike anywhere else in the world (McCann, 2017). So, although place may be evoked to resist local healthcare reforms (Moon & Brown, 2001), health and care is increasingly enrolled into place‐promotional activities, such as marketing private hospitals (Kearns et al., 2003) and inter‐place circulation of “exemplar” public health policies (McCann & Temenos, 2015).

Of particular interest to us here is the growing prominence of health and biomedical innovation within the remaking of “global” cities and regions. Singapore's developmental state‐led vision to become Biopolis of Asia, for instance, promotes the city‐state's “world class” biomedical cluster in a bid to attract global capital and clinical expertise (Singapore Economic Development Board, 2009). Established in the years following the 1997 Asian financial crisis, Biopolis brings together state agencies, public research institutes, and private companies within a signature‐architecture biomedical complex. Efforts to attract foreign direct investment are facilitated through financial incentives, strong intellectual property protection, and a supportive human biomedical research regulatory regime (Sparke, 2013, p. 316; Waldby, 2009). Not only does the “Singapore model” position biomedical experimentation as vital to the city‐state's economic competitiveness and the promise of future economic prosperity, it also seeks to align the governing of the biological life of the population with the biopolitical endeavours of the state (Waldby, 2009; Wong & Bunnell, 2006). In different ways, “big bioscience” is becoming dominant within global city strategies: from the enrolling of speculative biomedical science parks into city‐repositioning strategies in a bid to attract mobile capital, such as Guangzhou International Bio‐island, China (Xu & Yeh, 2005; Zhang et al., 2010) to developments promoting commercial discovery‐based “global scientific excellence,” such as the Francis Crick Institute in London, further inflating the “biomedical bubble” associated with the “golden triangle” of Oxford–Cambridge–London in South East England (Jones & Wilsdon, 2018).

There is, however, no guarantee such “global” visions will hold. As Sparke (2011) illustrates, health has been uneasily drawn into political struggles over the remaking of Seattle, USA, and its place in the world. Although its promotion as a competitive global city may indeed have become dominant, protests and social movements have pushed for Seattle to be re‐positioned as a very different kind of global city, resisting neoliberal globalisation and the World Trade Organization, while building collaborative networks to support various struggles for social justice. Differently again, Seattle has also been positioned as a “curative global city” in attempt to place it at the epicentre of global health philanthropy, forging links between citizenship, global health, and local economic development. While health initiatives have been increasingly pulled towards market‐friendly urban redevelopment strategies, Seattle is a reminder that the political construction of global cities and regions is always open to contestation and reinvention.

## 3 REMAKING GREATER MANCHESTER

The spaces of the British local state have transformed profoundly in recent decades (Cochrane, 1993; Jones, 2019). In this respect, Greater Manchester is no exception. With a population of 2.8 million, the city‐region of Greater Manchester in North West England is comprised of ten local authorities: Bolton, Bury, Oldham, Rochdale, Stockport, Tameside, Trafford, Wigan, and the cities of Manchester and Salford. The Greater Manchester (County) Council existed between 1974 and 1986, until metropolitan counties were abolished by the Thatcher‐led Conservative government following political hostility with municipal socialist urban councils opposed to pro‐market reforms. In Greater Manchester, the ten local authorities voluntarily formed the non‐statutory Association of Greater Manchester Authorities, enhancing joint working capacity and co‐ordinating strategic services across the metropolitan region. Having endured decades of de‐industrialisation, by the 1990s political leaders in the Labour‐led city of Manchester had taken a decidedly entrepreneurial turn (Peck & Ward, 2002). For instance, in bidding to hold the Olympic Games in Manchester, not only did Manchester City Council prove they could now talk the language of entrepreneurial growth to secure public grants, it also symbolised their ambitions to transform local governance and urban politics (Cochrane et al., 1996). Following a churn of regional initiatives in the years that followed, city‐regional agendas gained prominence across England, whereby Greater Manchester – with the city of Manchester at its political and economic heart – was increasingly presented as a “trailblazing” model for city‐regional governance (see further Deas, 2014). In this respect, Greater Manchester has come to be seen as rather exceptional.

Greater Manchester Combined Authority was established under the new Conservative‐led Coalition government as a statutory city‐regional body in 2011. It was the first of its kind in England. Although eight English core cities agreed City Deals with central government to drive economic redevelopment in 2012, Greater Manchester led another first by negotiating its own city‐regional “devolution deal” in 2014. The deal‐based approach would go on to typify the English devolution agenda (Ayres et al., 2018). Yet not only was the shifting of powers to the Combined Authority conditional on the election of a new city‐regional mayor, devolution was also inextricably bound to centre‐led austerity strategies characterising public policy since 2010 (Etherington & Jones, 2018; Gray & Barford, 2018).

City‐regional devolution was never solely a “national” vision. Narratives of Greater Manchester's history of joint working and pragmatic political leadership have long been used to lobby successive governments for increased devolved powers and resources (Lee, 2017). The Manchester Independent Economic Review was commissioned in 2009 by Manchester City Council, providing an intellectual basis for encouraging urban agglomeration to boost economic growth in Manchester and its surrounding city‐region (Haughton et al., 2016). A series of city‐regional growth strategies, such as the Greater Manchester Economic Review and the Greater Manchester Evidence Review, have since followed. Placing emphasis on “fiscal self‐reliance” in a decade dominated by austerity, New Economy – Greater Manchester Combined Authority's in‐house think‐tank/development agency – set out the long‐term vision for city‐regional growth and reform:

Our approach seeks to create a platform for fiscal self‐reliance in Greater Manchester … Creating jobs and growth without reforming services or transforming places will not reduce the costs of dependency. Economic inactivity amongst the working age population is one key cause of Greater Manchester's productivity gap. In order to maximise the benefits from the economic investment it is critical that there is investment to connect GM residents to that growth and to address both the productivity drag and to reduce the costs of public services. (2014, p. 3)

It was not until February 2015 that the separate health and social care “devolution” deal for Greater Manchester was announced. With initial terms of agreement set out in a Memorandum of Understanding approved by the Chancellor of the Exchequer (Greater Manchester Combined Authority, 2015a), the overarching strategic aims for the project were established in *Taking Charge of our Health and Social Care in Greater Manchester*, published at the end of the year (Greater Manchester Combined Authority, 2015b).

The vision was to achieve the “greatest and fastest improvement to the health, wealth and wellbeing of the 2.8 million people in the towns and cities of Greater Manchester” (Greater Manchester Combined Authority, 2015b, p. 2). Responsibility to close a £2bn “financial gap” by 2020/21 would be led by the new non‐statutory Greater Manchester Health and Social Care Partnership (GMHSCP), bringing together the ten local authorities, then‐12 NHS clinical commissioning groups (local purchasers of healthcare), 15 NHS (foundation) trusts (hospitals, mental health, and community services) as well as primary care and voluntary sector organisations. As part of the deal, GMHSCP negotiated access to £450m public funding intended strictly for “transformation” rather than reducing “deficits.” The complex governance arrangements would be overseen by a new Partnership Team with the Chief Officer employed by NHS England, an executive non‐departmental national public body. The new Greater Manchester Mayor did not gain overall responsibility for healthcare services, although public service strategies under the remit of the Mayor cut across health and social care. Despite the “devolution” branding, GMHSCP received more limited delegated functions on the promise of earning further devolved powers.

## 4 FOLLOWING THE POLICY

As with health and social care devolution, our approach to research was somewhat emergent. Shortly after announcement of the deal, members of the research team negotiated access for research allowing us to “follow the policy” (Peck & Theodore, 2012, p. 21). Working directly with NHS and local government managers across the city‐region, we would examine the devolution project as it unfolded. A series of research aims were agreed: to understand the overarching dynamics of devolution; to examine changes to the governing of health and care across the city‐region; and to trace changes to services. Notably, our research placed emphasis on learning from Greater Manchester.

“Policy is never a singular entity,” as Clarke et al. remind us, “it is put together – or assembled – from a variety of elements that are always in the process of being reassembled in new, often surprising ways” (2015, p. 9). Assemblage‐inspired research methodologies offer diverse, if contested, possibilities for critical policy and urban studies (Baker & McGuirk, 2017; Savage, 2019). Certainly, our adoption takes seriously the “concerns, concepts and analytical orientations of political economy” (Brenner et al., 2011, p. 232), yet does so by deploying a more methodologically open approach towards researching the making of policy and place. Within a research project funded on the basis of learning from a “unique” case study, our starting point was to trace through the multiple dimensions through which the devolution project was constructed, taking inspiration from studies of how “success” is produced and translated (Mosse, 2004), rather than necessarily beginning with a pre‐conceived analytical framework. The conceptual framing for this paper thus derives from “studying through” (McCann & Ward, 2012) the devolution project over two years, attentive to the multiple political forces, tensions, and competitive demands conditioning visions of becoming a global city‐region.

Given the emergent trajectories of devolution, we undertook what might therefore be termed a “distended” case study (Peck & Theodore, 2012, p. 21). Our approach harnessed in‐depth ethnographic research examining “local” socio‐political embeddedness while tracing connections and links across and beyond trans‐local sites, sensitive to the “global” circulations of policy. Recognising “policy‐making has to be understood as both relational and territorial; as both in motion and simultaneously fixed, or embedded in place” (Cochrane & Ward, 2012, p. 7), we studied transnational policy networking, circulating policy expertise and relations with “exemplars” identified elsewhere, without foregoing attention to more “inward‐facing” urban politics (Jonas & Moisio, 2018; McCann, 2013). Echoing Paasi, we sought “to step beyond simple dichotomies dictating that space should be understood as *either* territorially bounded *or* open” (2012, p. 2307, original emphasis), balancing ethnographic research across the city‐region with a sensibility towards how policies were arrived at through inter‐connections and relations with places and policies elsewhere, without reductively “fetishizing” the movement of policy (Robinson, 2015).

Working closely with GMHSCP, we undertook 50 interviews, principally with NHS and local government managers, as well as clinicians, management consultants, voluntary sector actors, and trade unionists. We observed 343 hours of meetings, mostly held outside public fora, involving hundreds of managers across various levels of seniority. Confidential papers and policy documents were received in advance of meetings. Like our research informants, we sought to read papers as thoroughly as possible beforehand. We became familiar with managers involved, occasionally travelling with them between meetings. Our study followed policy fieldtrips and networking conferences documented on social media and we attended devolution events organised by think‐tanks, unions, and universities, often including keynote speeches from GMHSCP managers.

As academics studying the “Greater Manchester experiment” based at a university with a keen interest in the “success” of devolution, we became public intermediaries entangled within the project we were studying. Careful attention is required to understand how researchers become attached to, and part of, policies under investigation (Temenos & Baker, 2015). Elsewhere, one of the authors has discussed the methodological implications of such unsettling “betweenness” as academic policy researchers (Lorne, 2021). It is important to emphasise here that we held frequent progress meetings with senior managers involved to discuss and reflect on research findings in “real time.” Annual policy roundtable and academic advisory meetings helped inform analysis. Research was formally presented to the GMHSCP at Executive level and policy‐focused interim and final reports assessing the initial impacts of health and social care devolution were published (Walshe et al., 2018). Findings relating to policy boosterism and competitive city‐regionalism are discussed for the remainder of the paper.

## 5 THE URGENCY OF DEVOLUTION: BETWEEN OPPORTUNITY AND CRISIS

We can't go any faster, but we can't afford to go any slower. (GMHSCP Manager, November 2017)

Health and social care devolution in Greater Manchester was hailed a “trailblazer” the moment it was announced (Greater Manchester Combined Authority, 2015a, p. 4). As the only place in England with such a deal, the city‐region courted intrigue and enthusiasm among policy commentators in the regional and national press. Not only was the scale of the project deemed “breathtaking” by the King's Fund think‐tank, but so too was the speed it was being pursued (BBC News, 2015).

Civic optimism and speculative opportunism marked the early stages. Local managers and political figures in Greater Manchester were able to deftly adapt to the compressed time‐scales required by national NHS bodies and central government (Peck & Theodore, 2015). For, if they were to realise their opportunity ahead, they would have to move quickly:

There was no template for this, so inevitably what we had to do was develop it as we went along based on our experience and contacts and ideas. So I think the main point I'll make is if researchers or academics who are searching for a very rational, evidence‐based, governance‐strong process, for how the transformation was initiated, how the strategic aims were initiated, will be disappointed, because a lot of what took place over that period was intuitive and opportunistic and driven by a pretty small number of key individuals, particularly in Greater Manchester but also some national people as well. (NHS manager, ID18; interview, August 2016)

When the deal became public, health and care integration appeared at odds with the privileging of competition with the market‐orientated *Health and Social Care Act 2012*. Policy running ahead of conventional routines now typifies the extra‐legislative dynamics of healthcare reform in England. It was not until a year later that the *Cities and Local Government Devolution Act 2016* would enable the possibility, at least, of formally transferring responsibilities and resources to the Combined Authority. Instead, if health and social care devolution was to “go live” by the start of the financial year in 2016, local managers would have to prove to both NHS England and the Treasury they could make it “work.” And quickly.

Various crises were required to be resolved through devolution. Greater Manchester and its ten localities were expected to “close the financial gap” of £2bn in five years, despite the lowest increase in NHS funding in its 70‐year history and cuts to local authority budgets that hit the north harder than almost anywhere else in England, directly impacting social care and public health services (Gray & Barford, 2018). Not only was pressure on Greater Manchester to transform rapidly, but there was seemingly no alternative:

We don't have a Plan B, we genuinely don't. (Local authority manager, ID62; interview, February 2017)

We often encountered frustration towards the impacts of austerity across the city‐region. Yet managers generally accepted if health and social care was to become “sustainable” financially and clinically, “radical transformation” would be necessary. Slidepacks were circulated illustrating the stark reality of the financial circumstances facing Greater Manchester alongside the savings required by 2020/1. Conditions for reform became characterised by the refrain “doing nothing is not an option.”

And yet pressure to reform in the face of crisis had a galvanising effect among managers involved. There was excitement about what might now be possible through devolution and a sense of civic pride among those keen to develop new place‐based approaches intended to improve health outcomes. Devolution was also seen to offer a chance to keep national regulators at a distance, while reducing the fragmentation and bureaucratic complexity in governing existing health and care services in response to perpetual national policy churn:

We are helped – is the wrong phrase – by the fact that the money's run out, you know? It's a crisis, it is a funding crisis for the NHS and there's been a funding crisis in social care which has been there for some time, and the transparency agenda around care with CQC [Care Quality Commission; regulator] and so on is contributing to that, so you can't sweep things under the carpet, which is quite right, and problems are being exposed. So, I think it's a combination of things coming together, and the appetite to say, do you know what, we'll give this a go. (NHS Manager, ID71; interview, August 2016)

With managers able to “capitalise on structural turbulence and uncertainty” facing health and care across England (Bailey et al., 2017, p. 215), Greater Manchester became a high‐profile policy experiment. Yet national regulatory bodies did continue to seek tight financial control and performance monitoring; the conditions for experimentation were constrained. The risk was, as Hodson and colleagues caution, “[b]y devolving responsibility for fixing problems without devolving resources, national government is pulling cities into a hyper‐competitive game whereby they must produce a narrative of innovation to get money” (2018, p. 1495).

Demonstrating quick “success” became vital for leveraging further devolved resources and powers. Considerable efforts were made to keep the city‐region together “as one,” such as allocating the £450m transformation funding in a way that would be seen to be “fair” for all ten localities. Yet, the health and care system across Greater Manchester was shaped through the interplay of local, city‐regional, national, and international actors and organisations coexisting, jostling, and forging uneasy alliances (see further, Lorne et al., 2019). For instance, although workforce protocols were signed, union representatives raised concerns about devolution. At a trade union event including speakers from GMHSCP, despite expressions of support for addressing regional economic unevenness and improving health, there was a worry devolution shifted responsibility for managing austerity to the city‐region amid uncertainty over how health and care integration would impact workers' pay, pensions, and rights. Frances O'Grady, General Secretary of the Trades Union Congress, had already affirmed “making devolution work” was a national priority issue, welcoming moves towards public service integration and increased collaboration between councils and the health service. Yet looking to Greater Manchester, she highlighted unresolved tensions warning “the ‘Northern Powerhouse’ rhetoric clashes with the reality of massive cuts to public services,” adding “there is the existential question about how to maintain the ‘National’ in a devolved NHS” (Trades Union Congress, 2016, p. 5).

Not long after, the rest of England had begun to embark on similar “place‐based” reforms (Hammond et al., 2017). Managers reflected on the “relentless” pace of devolution in Greater Manchester coinciding with a need to keep ahead of places elsewhere:

I think what the added factors with devolution are, something about pace, so because we're in the spotlight and we want to do things as quickly as we can, some of these discussions are happening more urgently and more explicitly than they would have done before … [so we were] sitting down with consultants to say, from your experience elsewhere, what would help us to fast‐track this because we've got to go fast with this. (NHS manager, ID18; interview, August 2016)

Undoubtedly, there was hesitancy over the role of management consultants brought into the project. But working together as a unified health and care system moving quickly was seen to be essential. For all the conditionality, devolution was said to be a once‐in‐a‐generation opportunity that must not be wasted. And as the Chief Executive of NHS England would repeatedly announce: “the eyes of the country are on Greater Manchester.”

## 6 LEARNING FROM ELSEWHERE AND A PLACE BEYOND COMPARE

Exceptionalist claims about what Greater Manchester may learn from elsewhere became strategically important for raising the profile of the city‐region. In March 2016, a year after the initial announcement, a Health Committee met to examine progress and discuss lessons learnt so far. It prompted the then‐Chief Executive of Manchester City Council to declare:

We have not only looked within the UK, we have looked beyond as well. We have tried to secure access to the best examples and I think the overall judgment that we have come to is that while there are lots of very, very good examples, they have tended to be in particular places around particular programmes and that what we have to do is develop our capability to do this at scale along the lines of our five programmes. Quite frankly, it did dawn on us, it has to be said – towards the end of last year in particular – there are no real examples where this has been done at this scale, certainly in this country and if not in other places in Europe. (House of Commons Health Committee, 2016; Q237)

The claim of no comparable examples of integrated health and care across Europe is rather bold, the exceptionalist rhetoric resonating with the earlier suggestion of having no blueprint to copy. This is, of course, not the first time local elites have enlisted a degree of myth‐making when promoting the city of Manchester and its city‐region (Haughton et al., 2016). Conceding there may be relevant examples of health and care integration to learn from, the insistence was that nothing was happening *at this scale*.

Playing with the “politics of scale” (Allen & Cochrane, 2007) through boosterist narratives helped local state managers negotiate tensions between the extrospective positioning of the city‐region at the global “cutting‐edge” with more introspective politics to persuade and sustain public support for devolution (McCann, 2013). It was put to us that more than 200 policy programmes were being assembled simultaneously across the city‐region, including personal health budget pilots, major reconfiguration of clinical services, and developing new digital health infrastructure. Despite suggestion there was nowhere comparable to learn from, the multitude of policy ideas adapted across Greater Manchester certainly came from somewhere.

Local Care Organisations (LCOs) became an important component of the plan for Greater Manchester to manage population health and encourage self‐care. LCOs are adaptations of Accountable Care Organisations (ACOs), lengthy contract‐based population health payment models designed to improve the quality of care while reducing expenditure, frequently associated with emerging US healthcare models (Fisher & Shortell, 2010). The triple aim of rolling‐out LCOs was to foster resilience and keep people healthy to avoid the need for hospital‐based services, to improve the quality of care through integration, and to achieve the all‐important financial savings to “slow the growing rate of public expenditure.” A multitude of state and non‐state intermediaries were involved in reworking and embedding ACO‐inspired models across the city‐region:

My first visibility of it was really reading [NHS England Chief Executive] Simon Stevens' piece in the *Five Year Forward View* which sort of rang true with ‘let's see how we can integrate and form more of a futuristic resilience in primary care.’ [Then‐Chief Executive, Manchester City Council] obviously got sight of the American programmes at that stage, and I don't know if you were present in the meeting, we had a meeting with Deloittes who then told us all the world systems very early on and talked us through them, and I remember it to this day because they told us the Canadian [model] … And then it was born really out of that. So, it's inspired by lots of different things. And we just called it LCO because ACO was too American I think, and we just wanted to own something that could mean anything at that stage. (NHS Manager, ID35; interview, April 2017)

As hinted above, circulating health policy models associated with the USA are politically contentious in England, framed as the “Americanisation” of universal healthcare by health campaigners. Managers carefully manoeuvred between presenting ACO models as “global best practice” while emphasising the localness of LCOs in each of the ten localities, all while reassuring Greater Manchester was not becoming wholly detached from a public *National* Health Service.

Initial exceptionalist claims mutated over time as local managers forged new transnational health policy learning networks. With conferences important arenas for circulating policy models (Cook & Ward, 2012), GMHSCP was regularly invited to showcase the “devo difference” at national think‐tank events and the annual NHS Expo conference, now held in the city of Manchester. Existing local government initiatives such as the “Wigan Deal” (Wigan Council, 2014) were shared widely as a local solution to managing austerity by combining social enterprise, civic empowerment, and self‐responsibilisation. Yet, the *global* ambitions for Greater Manchester became increasingly significant. Benchmarking with international comparators proved useful:

We are aware of relatively few examples in the world where this type of vision of a unified population health approach is being harnessed with an accountable care delivery system. There are some comparisons with the Scottish model of reform and also New Zealand. Another is the New York State Medic‐aid system reforms, particularly in their ‘best in class’ areas such as Staten Island. We have invited the core team in Staten Island to visit GM to demonstrate how you can combine great data analytics, community‐based population health programmes and integrated care to secure improved outcomes. (Public GMHSCP papers, March 2017

It was made clear, “our peers are not just in England, we're interested in understanding the international context” (NHS manager; meeting observations, May 2017). Policy networking would go on to help boost the status of the city‐region and attempts to become a “front runner” within a global “best in class.”

A study trip to New York State followed, with GMHSCP delegates tweeting their shared ambitions through twinning with their New York partners. In spite of the different political and funding arrangements for health and care, emphasis was placed on the common challenges they faced when moving towards holistic, “whole‐person” care. Reporting back, New York was described as “the holy grail” for transformation harnessing data analytics within population health management (NHS manager; meeting observations, June 2017). After a return trip to Greater Manchester, a senior manager from the New York State Medicaid programme later stated “Greater Manchester is a global epicentre for health and social care innovation … The world has much to learn from Greater Manchester and the local innovators who are transforming care for their entire population.” Cooperation and competition through policy twinning was thus strategically helpful in boosting Greater Manchester's international credentials (Jayne et al., 2013). Resonating with Seattle's positioning as a “curative global health city” (Sparke, 2011), transnational policy learning facilitated new dialogue to promote health improvement. Yet, as the Chief Executive of Health Innovation Manchester (an academic health science centre established to foster innovation) reflected: “it's not until you travel abroad that you truly realise the uniqueness of our offer and the international regard for our trailblazing entrepreneurship and developments” (Health Innovation Manchester, March 2018).

## 7 BECOMING THE BIGGEST EXPERIMENT IN HEALTH AND CARE ON A GLOBAL SCALE?

The potential to be seized through devolution was the opportunity to combine health and care reform with economic growth. With the ambition to improve health, wealth, and wellbeing, the dominant vision for a “sustainable” Greater Manchester aligned its “functional economic geography” with reduced dependency on public services (see above New Economy, 2014). Sifting through the evidence in an effort to align health and care with the wider vision for the city‐region (Haughton et al., 2016), the Manchester Independent Economic Review offered a series of “successful” international cities to compare themselves against:

Where is the stuff … which evidentially needs to be done if we're actually going to support this conurbation to be the sort of successful city‐region that we aspire it to be, in terms of international comparisons with the likes of a Seattle or a Munich or places like that? Those are places that were cited by Manchester Independent Economic Review comparators saying: you're the size of that but your productivity's not that. So how do you get to levels of productivity that those cities exhibit? Well, those places at one level have complete control. (Local Authority manager, ID52; interview, May 2017)

As a self‐proclaimed “vanguard of devolution,” Greater Manchester was increasingly promoted as being in a unique position to test their city‐regional plan for growth and reform: “the timing could not have been better,” even if it is to be “tested sooner than expected” (HSJ, 2015, pp. 16 and 17).

Forging new links between health and economic growth became more established over the course of our study. Diverging from more biomedical‐led competitive city visions (Waldby, 2009), the Chief Officer of GMHSCP emphasised their 20 “unapologetically evidence‐based” population health programmes targeting the social determinants of health. Framed through the prism of asset‐based community development (Roy, 2017), emphasis on local empowerment sat in tension with austerity politics:

We strive to make the very most of our total public service offer to equip individuals and communities to connect to the growing economic opportunities that this city region is generating. We want people to feel and to be empowered to take more charge of, and responsibility for, their own lives, to have more control and more choice to influence their own health and wellbeing … We are under no illusions that we are pursuing this Plan in the prevailing wind of austerity. Over the next five years we still have a financial gap of over £1 billion to close in terms of our NHS and social care pressures. To put it simply, when we took charge we were spending beyond our means by £200 every minute; if our plans don't work this will have risen to £2,000 a minute by 2021. (Chief Officer, GMHSCP, 2017)

Reasserting the urgency to reform, empowerment was not wholly situated as embedding neoliberal self‐responsibilisation, rather “poor health is holding back the Greater Manchester economy and a poor economy is holding back good health” (Health Innovation Manchester, 2018, np). Managers often acknowledged the long‐term impacts of economic restructuring on healthy life expectancy. Yet the balance of power was clear, with those working on the population health programme asserting “rebalancing the economy requires rebalancing public services” (meeting observations, 2017).

Health innovation and the life sciences had already been identified as a growth opportunity within the Manchester Independent Economic Review. This was crystallised following the devolution deal within policy documents such as the *Greater Manchester Internationalisation Strategy 2017–2020* citing health innovation as a global market to exploit in a bid to attract capital and a skilled labour market (Greater Manchester Combined Authority et al., 2017). Drawing closer with visions for Singapore as Biopolis, agreements with pharmaceutical companies were signed to develop Greater Manchester as a “globally competitive living lab” in health innovation. The *Financial Times* ran an article outlining enthusiasm within Greater Manchester to accelerate new forms of partnership with pharmaceuticals in rapid‐rollout population health initiatives. Greater Manchester's integrated care system was positioned as offering a territorially‐coherent “one‐stop shop” to gain access to a real‐time testbed of 2.8m people.

The “no Plan B” urgency of devolution became an opportunity to compete for health and biosciences R&D concentrated in South East England (Jones & Wilsdon, 2018). Having a “global” research‐intensive university on Oxford Road in Manchester was a strategic asset to boost competitiveness. A delegation of senior figures from academia, industry, and healthcare in Greater Manchester travelled to San Jose, San Francisco, and Boston to promote the city‐region with the aim of attracting heath innovation and MedTech companies. Labour leader of Manchester City Council, Sir Richard Leese elaborates:

With the strength of our world‐class universities in medical research, and having the UK's first integrated health and social care system, Greater Manchester is a highly‐attractive business destination for US companies. As the best city to live in the UK, our city region is a great place to study, work and invest. (2017, n.p.)

Having raised the international profile of the city‐region, the local state now hailed Greater Manchester as the biggest experiment happening in health and care on a global scale. Announcement of a new “world‐leading” precision medicine campus with global diagnostics firm QIAGEN soon followed, alongside expansion of the nearby CityLabs 2.0 and 3.0 “biomedical centre of excellence,” as part of a new vision for an Oxford Road Corridor Innovation District in Manchester.

Extrospective policy boosterism therefore became intertwined with the local growth coalition promoting their unique opportunity to attract mobile capital to the city‐region. Or rather, the city of Manchester:

It is undoubtedly true that Manchester is the heart of Greater Manchester, and Greater Manchester needs Manchester to succeed if Greater Manchester is to succeed. But I would say that's the case economically, jobs, prosperity wise, etc. as well as in health and social care. So, there's a grudging respect and acceptance of that I think from other parts of GM, and that sometimes bubbles up into frustration, but that's the accepted reality. (NHS manager, ID18; interview, August 2016)

Here, tensions surface with introspective urban politics and the dominance of trickle‐down, trickle‐out agglomerative growth strategies. Since 2013, austerity‐stricken local authorities in England have again become responsible for public health. With central government pushing for local authorities to become financially self‐sufficient by moving towards 100% business rate retention to fund services, the spatialised conflicts inherent to aligning a “globally orientated” competitive economic growth agenda with “local” management of health and care becomes apparent. As a manager cautioned:

Sexual health services, health visiting services, school nurse services, really important clinical NHS services … is on a trajectory to be cut and cut, and then replaced by retained business rates. And that's actually a fundamental change to how the NHS is funded … [with] some of our NHS funded through business rates … if you live in Manchester, you're alright actually. But if you live in Rochdale and Oldham, and places where they have less business rate [revenue], you know, then there's a double whammy coming along isn't there for those [local authorities]. (Local authority manager, ID2; interview, February 2016)

Political commitments were later made to promote “inclusive growth” across the city‐region. Attempts to embed “social value” within local commissioning activities were pursued. And although the orthodoxy of an agglomerative economic growth model has since witnessed increasing challenge in struggling towns such as Oldham, the “successful” Manchester‐centric model prevails, and with it the familiar risk of a “race to the bottom” to attract private investment (see further Deas et al., 2020).

In little over three years, the new Greater Manchester Mayor, Andy Burnham, had moved away from critiquing health and social care devolution as creating a two‐tier NHS to championing a new “*Greater* Manchester model.” The city‐region could now embed health and care across all economic and social policies:

To rise to today's challenges, the NHS needs a change of thinking as seismic as the creation of the NHS itself … as Mayor of the only city‐region with health devolution, it has become increasingly clear to me that the unique opportunity Greater Manchester has is to integrate health with everything – early years, education, community safety, housing and employment … Greater Manchester has a major opportunity to become a global centre for investment in life sciences and digital – where new concepts can be tested and proven in real‐time and real‐world environments. (Burnham, 2018, n.p.)

Having met with mayors from around the world, he assured the evidence was now clear: global city‐regions are the future, as *the* best scale for organising economic growth and public services (Harrison & Hoyler, 2014). Devolution might even be as transformative as the creation of the national welfare state. For, not only did he insist Greater Manchester was “doing things differently,” their pioneering, competitive model was also pushing the city‐region “further, faster” (Burnham, 2018).

## 8 CONCLUSIONS: PUSHING THE CITY‐REGION FURTHER, FASTER

Our aim in this paper has been to examine the promotion of health and care policy within the political construction of competitive city‐regions. Responding to the need to understand how city‐regional institutions, actors, and spatial imaginaries are enrolled into new forms of geopolitical experimentation and problem‐solving (Jonas & Moisio, 2018), we have expanded McCann's (2013) work on policy boosterism to trace the relational and territorial geographies of policy across and through new “devolved” city‐regional arrangements in Greater Manchester. Following Allen and Cochrane (2007), we have demonstrated how local state managers harnessed health and care policy to negotiate a more fluid politics of scale, fostering overtly transnational policy learning networks while simultaneously hailing the “world‐leading” city‐region as a pioneer “doing things differently.” From hospitals and universities to inward investment agencies and the city‐regional mayor, health and social care integration was embedded within the local state's internationalisation strategy premised predominantly on “trickle‐down” agglomerative economic growth. Rather than solely being at the receiving end of devolutionary austerity, we have shown how efforts to resolve the “local problem” of governing health and social care under austerity were rearticulated as a new “global opportunity” in an attempt to achieve the greatest and fastest improvement in health, wealth, and wellbeing.

City‐regions have become powerful spatial constructions in recent decades, with debates far from settled as to how best to analyse their ongoing reinvention. Certainly, the study presented here takes a view from somewhere; like many of our research informants, we hold a deep commitment to place. And yet, while the local state may well be compelled to privilege narratives of exceptionalism, we face analytical questions as to whether we, too, can situate the city‐region beyond compare (Peck, 2015). We concur with McCann and Ward (2010), as studying the contradictory dynamics of policy and place offers a productive lens for understanding the remaking of city‐regions. Advancing a conceptual framework linking crisis and opportunity, emulation and exceptionalism, and evidence and experimentation, we have sought to move beyond an impasse in recent scholarship on the political construction of city‐regions by holding in tension different territorial and relational geographies within our analysis. Studying through a seemingly unique city‐region allows us to examine how health and care policy is being pushed in new, experimental directions, while bearing uncanny resemblance with places elsewhere.

So, if we are facing a decisive moment in global capitalism, then what is the future role of health and care for city‐regions in pursuit of international competitiveness (Jonas & Moisio, 2018)? Does the enrolling of health and care into global city‐region strategies really mark a transformation as “seismic” as the formation of the Keynesian welfare state? Despite the boosterist claims, the reconfiguring of powers to Greater Manchester were never quite as radical as portrayed by either its advocates or detractors. Nonetheless, health and social care policy was reworked by the local state in a global race to attract mobile capital by championing “global excellence” in health and biomedical innovation (Wong & Bunnell, 2006). Yet diverging from visions of the Biopolis governing through biopolitical experimentation (Waldby, 2009), the city‐region's population is now the target for health and wellbeing strategies through self‐responsibilisation with an aim to boost economic productivity while slowing growth in public expenditure. Such approach resonates with the rapid uptake of Health in All Policies and Healthy Cities initiatives now championed by agencies such as the World Health Organization (Naylor & Buck, 2018). Yet drawn into the competitive logics of urban entrepreneurialism (Harvey, 1989) – intensified by cuts to local authority budgets – such interventions risk deepening already‐existing social divisions (Cole et al., 2017). Comparative research examining social and health inequalities within and between global cities and regions touting good, healthy competition is undoubtedly warranted. And crucially, as Sparke (2011) reminds us, there is no guarantee such “global” visions will hold.

The geographies of the global city‐region gained rather different significance following the outbreak of the COVID‐19 pandemic. Looking out through the lens of Greater Manchester, the current crisis is condensing tensions and contradictions embedded within English devolution. At a time when many of the architects of Greater Manchester devolution have since departed, responses to the pandemic have been resolutely undermined by central government pursuing massive public health outsourcing to the private sector, despite suggestion of initially responsive local coordination (HSJ, 2020a). Yet with the city‐regional “experiment” set to be reviewed and redesigned, the Greater Manchester Mayor has since conceded “there may have been some unrealistic expectations of what it could deliver” (HSJ, 2020b, np). With a catastrophic number of deaths across the UK, poorer towns in Greater Manchester such as Oldham and Bolton have had some of the highest rates of infection in England. How health vulnerabilities are structured by racial violence and antiblackness (Hirsch, 2020) and class inequalities compounded by the uneven effects of austerity (Dorling, 2019) demands urgent and sustained political and academic response. Speculative luxury apartments have transformed Manchester in recent years, yet the city‐region faces a growing housing crisis (Silver, 2018). Forced to become increasingly financially “self‐sufficient,” several councils in Greater Manchester may soon be declaring themselves effectively bankrupt (Manchester Evening News, 2020). Pulling health and care towards the coercive dynamics of inter‐urban competition premised on trickle‐down, trickle‐out agglomerative growth risks ignoring growing intra‐urban spatial unevenness despite ambitions to foster “inclusiveness” (Beel et al., 2016; Deas et al., 2020). With growing attention towards the social determinants of health within visions such as Healthopolis, we would be wise not to forget Manchester's place in foregrounding the links between capitalism, bad living and working conditions, and poor health (Engels, 1999 [1845]).

Connections forged between health, place and economy are once again being re‐imagined in the present crisis. To talk of crisis and contradiction, we do not seek political economic reductionism for understanding the current moment. But we certainly don't suggest abandonment. The pandemic situates health and social injustices within deeply entrenched geographies of class, race, and gender inequalities. Yet it has also re‐established the vital importance, celebration even, of health and care workers within the public imagination, alongside new nation‐building claims. As yet another economic recession begins to set in, what the future holds for health and care policy within competitive city‐regional strategies is decidedly unclear. Certainly though, future research into the unstable relations between capitalist economies, welfare states, and health and wellbeing necessitates critical dialogue beyond disciplinary boundaries. Perhaps, then, keywords of “articulation” and “conjuncture” will prove vital in thinking through the geographies of crises and the ongoing reconfiguring of global city‐regions in good health and turbulent times (Clarke, 2019; Hall & Massey, 2010). Here, we provide direction by emphasising the accumulating forces, tensions, and contradictions when turning to health and care to push the city‐region further, faster.

## Data Availability

Research data are not shared.

## ENDNOTES

^1^ With thanks to one of the reviewers for neatly describing policy boosterism as a “vector” of competitive city‐regionalism.

^2^ Unlike (most) NHS health services in England, social care is needs‐ and means‐tested. However, NHS charges for overseas visitors regulations have been in place for many years and an “immigration health surcharge” was recently introduced as part of the UK government's “hostile environment” policies.
